# Supplementary material for: The epidemiological and economic burden of diabetes in Ghana: A scoping review to inform health technology assessment
Source: PLOS Glob Public Health. 2024 Mar 12;4(3):e0001904. doi: 10.1371/journal.pgph.0001904 (PMC10931482; doi:10.1371/journal.pgph.0001904)
Supplement: S1 Text — (DOCX) [file pgph.0001904.s002.docx]

**Supplementary file 1: Search strings**

**Web of Science search**

Search link for Web of Science

<https://www.webofscience.com/wos/woscc/summary/55ba5b67-9492-4899-a3dd-8b2fe3588006-80f3249d/relevance/1>

(TS=(Ghana)) AND TS=(Diabetes)

**PubMed search string**

(("ghana"[MeSH Terms] OR "ghana"[All Fields] OR "ghana s"[All Fields]) AND ("diabete"[All Fields] OR "diabetes mellitus"[MeSH Terms] OR ("diabetes"[All Fields] AND "mellitus"[All Fields]) OR "diabetes mellitus"[All Fields] OR "diabetes"[All Fields] OR "diabetes insipidus"[MeSH Terms] OR ("diabetes"[All Fields] AND "insipidus"[All Fields]) OR "diabetes insipidus"[All Fields] OR "diabetic"[All Fields] OR "diabetics"[All Fields] OR "diabets"[All Fields])) AND (2021/4/4:2023/4/11[pdat])

**Embase search string**

('ghana'/exp OR ghana) AND ('diabetes mellitus'/exp OR 'diabetes mellitus')

**Scopus search string**

(TITLE-ABS-KEY ( ghana ) AND TITLE-ABS-KEY ( diabetes ) ) AND PUBYEAR > 2020 AND PUBYEAR < 2024
